# The Potential of the Remote Monitoring Digital Solutions to Sustain the Mental and Emotional Health of the Elderly during and Post COVID-19 Crisis in Romania

**DOI:** 10.3390/healthcare11040608

**Published:** 2023-02-17

**Authors:** Marilena Ianculescu, Adriana Alexandru, Elena-Anca Paraschiv

**Affiliations:** 1 National Institute for Research and Development in Informatics, 011455 Bucharest, Romania; 2 Faculty of Automatic Control and Computer Science, Politehnica University of Bucharest, 060042 Bucharest, Romania; 3 Faculty of Electrical Engineering, Electronics and Information Technology, Valahia University of Targoviste, 130004 Targoviste, Romania; 4 Faculty of Electronics, Telecommunications and Information Technology, Politehnica University of Bucharest, 060042 Bucharest, Romania

**Keywords:** Remote Monitoring Digital Solutions (RMDSs), elderly patient, mental and emotional decline, COVID-19

## Abstract

The COVID-19 pandemic amplified the elderly’s aging-related dysfunctionalities and vulnerabilities. Research surveys, aimed at evaluating the socio–physical–emotional state of the elderly and obtaining data on their access to medical services and information media services during the pandemic, were carried out on Romanian respondents aged 65+. Identification and mitigation of the risk of emotional and mental long-term decline of the elderly after SARS-CoV-2 infection, based on the implementation of a specific procedure, can be performed through Remote Monitoring Digital Solutions (RMDSs). The aim of this paper is to propose a procedure for the identification and mitigation of the risk of emotional and mental long-term decline of the elderly after SARS-CoV-2 infection that comprises RMDS. The importance of using the knowledge obtained by COVID-19-related surveys corroborating the necessity of including personalized RMDS in the procedure is highlighted. The Non-invasive Monitoring System and Health Assessment of the Elderly in a Smart Environment (RO-SmartAgeing) is an RMDS designed to address the improved preventative and proactive support for diminishing this risk and to provide suitable assistance for the elderly through a safe and efficient smart environment. Its comprehensive functionalities targeted supporting primary healthcare assistance, specific medical conditions—as the mental and emotional disorders post-SARS-CoV-2 infection—and enlarged access to aging-related information, together with customizable features, illustrated the match with the requirements included in the proposed procedure.

## 1. Introduction

SARS-CoV-2, the virus that causes coronavirus disease 2019 (COVID-19), spread in China in early December 2019. On 12 January 2020, the World Health Organization (WHO) confirmed that COVID-19 was the cause of respiratory illness in China [1], having a case–death ratio much lower than that of SARS in 2003 [2], but a notably greater transmission, with an important total dying rate [3]. The virus affected more than 250 million people globally, with more than five million deaths having been reported by the WHO [4]. The virus spreads human-to-human, causing flu, fever, cough, and respiratory problems [5,6]. The prevalence of the virus has been prevented through constant lockdowns, which highly affected the economy and small businesses [7,8]. In order to stop the pandemic, self-quarantine in their residences was enforced on normal people, and quarantine in hospitals until full recovery was enforced on people affected by the virus.

To better understand the pandemic and the impact of COVID-19 on healthcare services in Romania, a short overview is provided: the actions to prevent the spread of the pandemic started in mid-March 2020 (e.g., interdiction of public gatherings, school closure). A state of emergency in Romania was decided, starting on 16 March 2020 [9]. In mid-May 2020, some restrictions were relieved when the state of emergency ended on 14 May 2020 and was changed to a state of alert. Further restrictions were relaxed in the next months. Since an acute increase in the infection rate was observed in the autumn of 2020, several restrictions were reinforced. Schools were re-opened only on 8 February 2021. Between mid-March 2021 and the beginning of March 2022, three waves of cases were noticed in Romania. The wave of cases between 10 August and 20 October 2021 has been the most severe of all infection waves in the COVID-19 pandemic. The pressure on the Romanian healthcare system that achieved its maximum capacity in this wave led to the authorities asking the European Union for help through its Civil Protection Mechanism [10].

Older people were highly affected by COVID-19, and many of them have died [11]. This is due to their low immunity, which is insufficient to fight against the virus. As the risk of mortality in the older population is much higher compared to younger people, protection with social distancing or, if necessary, social isolation is necessary. Loneliness and social isolation may cause many problems, such as mental and emotional problems, disability, cardiovascular diseases, etc., among seniors [12]. The ongoing COVID-19 pandemic has underlined the need for digital technology solutions to diminish the risk of contamination due to close contact [13]. Smart technology (e.g., IoT, mobile phones) has been important for diminishing social isolation, improving the quality of life and self-care, and providing consultation, remote monitoring, and diagnosis for older adults [14].

The numerous restrictions imposed by COVID-19 in many countries led to a damaging effect on the psychological functioning of the elderly [15], loss of social support due to limited contact with other people [16], higher levels of loneliness, which is significantly associated with depression in the elderly [17,18]. Loneliness is considered to be an important risk factor for the exacerbation of a number of health conditions, such as coronary heart disease and stroke [19], and is associated with a 26–50% increased risk of mortality [20].

There are several instruments for assessing the mental and emotional health of the elderly during and post-COVID-19 crisis. They are used to measure her/his cognitive impairments and to screen for dementia or mild cognitive impairments (MCI) for estimating their severity and progression or for following the course of her/his cognitive changes over time. These instruments are used as screening tests, but they need to be used with multiple other screening tests, rather than an isolated one, to confirm a diagnosis of dementia. Some of them are presented below:

- The *Mini-Mental State Examination (MMSE)* or *Folstein test* is the best-known and the most frequently used screening tool for estimating the cognitive impairment of an individual. It consists of a 30-point questionnaire which takes 7–8 min to complete. Using the MMSE over time, one could better predict the conversion to dementia from MCI stages for people with more pronounced symptoms [21].
- The *Montreal Cognitive Assessment (MoCA)* test for dementia is a brief, simple, and reliable tool to evaluate people with memory loss or other symptoms of cognitive decline or a screening tool for conditions such as Parkinson’s disease, brain tumors, and head trauma. It consists of a 30-point questionnaire which takes 10–12 min to complete (taking a few minutes more than MMSE). The MoCA may be a better choice for people with mild symptoms than MMSE. The MoCA test checks language, memory, visual and spatial thinking, reasoning and orientation skills, and executive functions, and implements the clock exercise (that the MMSE does not have). Using it, healthcare providers can quickly decide when someone might need an in-depth diagnostic for Alzheimer’s disease or dementia [22].
- *Mini-Addenbrooke’s Cognitive Examination (MACE)* is a brief cognitive screening instrument for the identification of dementia and MCI [23]. It is comparable to MoCA, being rapid, accurate, easy to use, and well accepted by seniors. MACE and MoCA are excellent for dementia diagnosis (both >0.9), but NACE has a slight net benefit on MCI diagnosis [24].
- The term *Activities of Daily Living (ADL)* is used to describe the basic skill necessary for caring for an independent living by oneself. (eating, bathing, mobility, dressing, continence, etc.). ADL indicates the functional status of an individual. It is used as a predictor of functional status deterioration and possible necessity of hospitalization, other living arrangements, or assisted home living. Aging can cause a decline in the functional status of seniors and is the principal cause of ADL damage [25], leading to decreased physical functions. A cognitive or mental decline [26] or social isolation can be associated with impaired engagement in ADL.
- The *Free-Cog* test is a hybrid cognitive screening instrument for assessing both cognitive and functional (executive) functions. It combines questions in both domains: for cognitive deficits, it uses questions related to orientation in time and place, memory, calculation, attention, visuospatial function, language, fluency, etc. (in a 25-point questionnaire), and for functional ones, it uses questions related to daily activities including social functioning, travel, self-care, and safety at home (in a 5-point questionnaire) [27].
- The *Mini-Cog* test is a fast and simple screening method for early detecting the first stages of dementia. It consists of two components: a three-item test for memory evaluation and a clock drawing test. As it can be completed in approximately 3 min, it is often used to detect early stages of dementia in which memory or thinking issues might not be that visible. It proved to be appropriate for use in primary care evaluation [28].

The entire impact of the COVID-19 pandemic on the wellness and health status of the elderly has not been completely assessed; what is certain is that this impact has been perceived keener among the older persons, taking into consideration all the restrictions imposed by the authorities (including the lockdowns) in their attempt to reduce and control the degree of contagion. Moreover, isolation was also imposed on the elderly by their family and friends, most of the time to protect them from getting ill.

A study performed on 254 persons who were hospitalized in Italy due to SARS-CoV-2 infection revealed that the elderly patients with persistent psychiatric and somatic symptoms perceived these symptoms more strongly in the following six months post-infection, followed by a decrease afterward [29]. Another study performed at Daping Hospital in Chongqing, China, on COVID-19 patients aged 60+ stated that 21% of those who had had a severe form of the disease suffered from progressive cognitive decline afterward; the more severe the SARS-CoV-2 infection, the higher the risk of deterioration of mental health [30].

It is almost a general fact that nowadays, the elderly are much less tech-savvy than the younger generations, which directly leads to fewer possibilities for them to have access to information (including medical information) or social interaction; another element that has to be taken into consideration is the lower number of digital devices used by the elderly, as they might be too expensive or not age-friendly enough. It is not to be neglected either the situation in which an exacerbated access to scientifically unverified and alarmist information, combined with social isolation, leads to increased anxiety, depression, and other emotional and mental problems. So, the lockdowns and restricted access to the out-of-home world imposed in different periods of time since the beginning of the COVID-19 pandemic had a strong negative effect on the mental and emotional state of older persons.

Some typical mental health disorders that have been estimated to directly affect people post-SARS-CoV-2 infection are as follows: anxiety, panic attacks, depression, dietary and obsessive–compulsive disorder, personality disorders, paranoia, phobias, psychosis, sleep problems, and suicidal thoughts.

The elderly have been among the most vulnerable group of the population from the point of the mortality and serious physical damage of health status directly associated with SARS-CoV-2 infection; it has already been demonstrated that it is no less true that the degree of morbidity, mortality and accelerated mental decline has increased among them due to social isolation, grown stress and the decrease in cognitive provocation and physical activities.

Since the beginning of the COVID-19 pandemic, a series of surveys have been conducted in Romania regarding how it has affected, especially from a mental and emotional point of view, the population at the global level, but especially the elderly. The isolation and fear of contamination with the SARS-CoV-2 virus, the fear of death, loneliness, the limitation/lack of access to medical services, the accentuation of pre-existing mobility and health problems due to loneliness, as well as the limitations imposed on social life, have affected, in particular, people aged 65+. This was also demonstrated by the surveys carried out by (1) the Romanian Institute for Evaluation and Strategy (IRES)—an independent think tank that conducts surveys on the problems and perceptions of Romanians on current issues; (2) Kantar Romania—a data and evidence-based company that operates in the Marketing Research and Public Opinion Polling sector and provides insights and actionable recommendations to its clients—at the request of the Never Alone Association—Friends of the Elderly—an NGO that supports the cause of the loneliness of the elderly and promotes dignity at any age. The methodology used in such surveys, as well as their results obtained at various times during the COVID-19 pandemic, are presented in the paper.

In order to identify and reduce the effects of the COVID-19 pandemic on older adults (such as emotional and mental decline), the authors propose a procedure for managing the associated risk through RMDS.

Due to the short time since the beginning of the COVID-19 pandemic, no solid assessment of the effects on the mental and emotional state of the elderly following SARS-CoV-2 infection has been performed. Even less, no reliable methodologies have been implemented by which these negative effects, which can significantly influence the health of the elderly in the long term, can be managed, controlled, or annihilated.

As there is a continuous growth of RMDS at the national and international level, in Romania, there are several research gaps in this regard. The lack of correlation between the screening tests for neurodegeneration and evaluating patients’ medical data is crucial, as healthcare parameters have an essential role in assessing neurodegeneration. Additionally, considering the fear of contamination with the COVID-19 virus, there are not considerable systems that can remotely monitor the elderly in the comfort of their home. The lack of access or the difficulty in reaching medical services related to prior mobility or healthcare issues composes another gap in providing a good quality of life for the elderly. Moreover, there is also the absence of a complex platform that could not only track health parameters, but also sustain mental or behavioral problems.

According to the above-mentioned gaps, the present research paper aims to meet all the needs that can provide a complex and structured system not only to remotely monitor health parameters, but also to track and screen neurodegenerative-associated patients and sustain them emotionally and socially.

The relevance of such solutions during the pandemic is important in managing older people’s wellbeing and mitigating the pressures on the healthcare system [22,23,24,25,26,27,28,31,32,33].

The addressed population mainly consists of older people in need of communication, with mental health problems, and suffering from social isolation [34]. The RMDS has been largely used in the last two years [35,36] for dealing with social isolation and loneliness [37,38,39] and mental health consultation purposes [40,41] during the crisis. With the help of m-health tools, information [42,43] and self-help [44,45] have been provided to the elderly. Internet access [46,47] and the senior’s desire and capabilities [32] are the main factors in the success of such solutions to improve their health and combat the COVID-19 outbreak. Some of the facilitators in the adoption of these solutions are the support from the government and family [33,48,49].

In the context of the COVID-19 pandemic, the Non-invasive Monitoring System and Health Assessment of the Elderly in a Smart Environment (RO-SmartAgeing) is implemented as a tailored patient-centric RMDS aiming at assessing and the health management of older patients that are living at home. While its two main components include quite a large range of functionalities able to address primary healthcare issues and support for healthy, independent, and active aging, the potential mental and emotional disorders of the elderly can be identified, evaluated, and tackled both by the elderly, and their supporting people, including the medical staff. The system gathers health, motion, and environmental data from IoT sensors and devices, transmits them, and stores them to the cloud platform for advanced data analysis applications. The IoT-based sensors and devices are programmed to send the data, via an Application Programming Interface (API), to the RO-SmartAgeing database and can be further visualized and analyzed in the web platform. The services provided to the seniors consist of medical assistance and support provided by using a safe, customizable smart environment. The beneficiaries of such services are older persons, their current healthcare specialists, and caretakers/family [50].

As the COVID-19 crisis had such a dramatic impact on individuals and the societal, medical, and social systems in Romania, the development and implementation of digital solutions and technology targeted to support remote health monitoring of the elderly have been accelerated, and gained larger accessibility among citizens and health professionals. Moreover, the research in this field has been boosted, and digital healthcare solutions have been developed or improved to sustain remote care of the elderly, as briefly illustrated in Table 1.

The list of abbreviations used in this paper is presented in Table 2.

The aim of this paper is to propose a procedure for the identification and mitigation of the risk of emotional and mental long-term decline of the elderly after SARS-CoV-2 infection that comprises RMDS. The necessity of taking into consideration the results of COVID-19-related surveys and the implementation of appropriate RMDS into this procedure is presented and justified. The RO-SmartAgeing system is an illustration of an RMDS for addressing improved preventative and proactive support for diminishing this risk.

The content of the paper is organized as follows: the methodology used to estimate the effects of the COVID-19 crisis on a sample of the Romanian aged population, obtained from some surveys, is presented in Section 2.1. Issues that can sustain the management of the elderly’s risk of emotional and mental long-term decline after SARS-CoV-2 infection and the steps of a procedure for managing the elderly’s risk of emotional and mental long-term decline after SARS-CoV-2 infection are proposed in Section 2.2. The importance of involving RMDS in the management of seniors’ health and the steps for implementing functionalities provided by them are presented in Section 2.3. The effects of the pandemic on representative samples of the Romanian elderly, obtained as results of some surveys, are presented in Section 3.1. The results of this approach are described in Section 3.2.1 and Section 3.2.2 by using the capabilities of the RO-SmartAgeing system to address preventative and proactive support for diminishing this decline. Section 4 is a discussion section dealing with the presentation of the main findings of the paper, strengths, drawbacks, as well as lines for future work. Section 5 concludes the paper. The references are included at the end of the paper.

## 2. Materials and Methods

### 2.1. Effects of COVID-19 Crisis on a Sample of Romanian Aged Population—Results of Methodology Used in Some Surveys

According to the data published by the National Institute of Statistics (INS) on the International Day of the Elderly, on 1 January 2020 [57], people aged 65 and over in Romania represented 19% of the country’s resident population. Among the elderly, men totaled 15.7% of all men resident in Romania, and women 22.1% of all women resident in Romania. The segment of the population aged 80 and over represented 4.8% of the total population. Of the total number of people aged 80 and over, 34.8% were men, and 65.2% were women. The household structure based on the occupational status of the head of the household shows that pensioners represent 40.9%

The share of the population over 65 years of age in Romania, according to the statistical data from the 2022 Census published by the National Institute of Statistics (INS) [58], represents almost 20% of the total population.

Thus, according to the INS, the demographic aging process has deepened in recent years, noting the increase in the share of the elderly population (aged 65 and over).

The perceptions of Romanians regarding the COVID-19 pandemic have been constantly evaluated, starting from March 2020; until now, more than 30 studies have been carried out focusing on the way in which the life of Romanians, but also of the society perceived as a whole, was affected, in different aspects, by the pandemic. We briefly present the methodology of three of these studies and present aspects related to the influences of the COVID-19 pandemic on a sample of population aged 65+. Sample type was simple, random population satisfying the following criteria of eligibility for participation in this study: (i) people currently residing in Romania (aged 65 years and above) of either gender, (ii) comprehension of this study’s goal, and (iii) consent to participate in this study.

The research study included a qualitative part (in-depth interviews with people from the representative age segment) and a quantitative part (survey based on Computer Assisted Telephone Interviewing among the elderly) for a national representative sample. A well-structured, closed-ended, and self-reported questionnaire was created, including questions about COVID-19-related characteristics and perceptions. Each survey takes approximately 10–15 min to finish. Prior to the interview, study participants provided verbal consent over the phone. Participants were thoroughly instructed on the process and purpose of this study and agreed that their information would be kept confidential and anonymous. Participants were not compensated for their participation in the research and were free to withdraw at any moment without providing evidence. Those who refused to consent were not authorized to participate in the survey.

A sample of questions used in the three analyzed Romanian surveys is illustrated in Table 3.

### 2.2. Managing the Elderly’s Risk of Emotional and Mental Long-Term Decline after SARS-CoV-2 Infection

Among the most important issues to be identified in order to define a procedure for better management of the emotional and mental long-term decline after SARS-CoV-2 infection is to determine what the most relevant risk factors considering the specificities of the lifestyle and age-related dysfunctionalities of the elderly are. Since the beginning of the COVID-19 crisis, data and information have been collected or become available from local, regional, national, or international surveys or studies based on surveys from self-reports of the patients infected with SARS-CoV-2 [62]. For a better foundation, research and studies that correlate the medical history of elderly patients with new cognitive symptoms and disorders appeared after the SARS-CoV-2 infection started to develop. Special attention is paid to risk factors before and after infection. For instance, in a recent study addressing the long-term sequelae clustering phenotypes for appropriate care management post this type of infection, associated risk factors have been identified and classified [63].

In this context, the most relevant identified *risk factors* are as follows:

- The degree of autonomy and independence of the older person;
- The residential status: living alone or not;
- The previous and current state of cognition, health, and co-morbidities;
- A low degree of self-esteem, social involvement, and commitment;
- A low level of previous education, including ICT and health literacy;
- The lack or little physical activity and social interaction;
- The sex (28% of senior women in U.S. developed mental disorders during COVID-19 pandemic in comparison with 20% of senior men [64]);
- The age (26% of seniors aged 65–77 in U.S. declared mental disorders versus 19% aged 80+ [64]).

The level of impact on the elderly that these risk factors might have depends directly on both their medical history and cognitive and social status, but also on their adaptability and self-sufficiency.

In order to mitigate the psycho-social and mental wellness impact of SARS-CoV-2 infection on older persons, some basic actions must be performed/initiated:

- Early identification of the first signs of decline in mental and emotional health of an elderly person, corroborated awareness and precise identification of risk factors;
- An accurate and timely diagnosis of mental and emotional disorders;
- Proper management of the identified disease, integrated with the management of the health status of the elderly;
- Comprehensive analysis of mental and emotional health;
- Appropriate longitudinal studies for assessing the long-term efficiency of the above-mentioned actions;
- Tailored framework adapted to the specificities of the elderly persons that can provide them support for having access to reliable information associated with healthcare and daily living;
- Taking into consideration their long-life experience, older patients facing mental and emotional disorders can be constantly sustained by medical specialists or family members to cope with their fears, anxieties, mental blocks, or other cognitive issues.

In the context of the COVID-19 pandemic, *RMDS* proved to be a progressive model of care, as they can ensure, in the safest way, continuous monitoring and management of the health status. The elderly patients have been one of the most important potential target users of RMDSs; because of the imposed restrictions and isolation, these solutions for providing healthcare have been perfectly capable of creating a personalized framework in which an older patient is observed, consulted, and monitored non-invasively, constantly, or periodically, depending on the evolution of their state of health. Thus, unnecessary hospitalization or direct human contact in clinics or medical offices could be diminished.

In terms of monitoring and managing the mental and emotional disorders activated or aggravated by the COVID-19 pandemic, in two years, RMDSs have been able to demonstrate how their functionalities could support adaptive behaviors, social relationships, informational necessities, engagement in usual daily activities, increased self-abilities to cope with cognitive issues, and direct real-time link among healthcare providers, patients, and their families.

It is now time to step forward toward proper management of the risk of the decline that those elderly patients with mental and emotional disorders—that were influenced by the SARS-CoV-2 infection—may have in the long term. Moreover, the opportunities and benefits in this domain brought by ICT in healthcare domain should be reflected by integrating the capabilities of RMDSs among different levels, phases, and activities of this management.

#### Proposed Procedure for Managing the Risk of Mental and Emotional Long-Term Decline during and after SARS-CoV-2 Infection

Although there are many strategies and procedures recommended by health authorities and specialists for decreasing the cognitive decline of the elderly, taking into consideration the short period of time since the beginning of COVID-19 pandemic, there are not many risk management plans addressing this domain in an integrated way, comprising comprehensive aspects that define the state of cognitive health and its evolution. Most of the strategies, procedures, and programs that aim to manage the mental and emotional status of the elderly refer to recommendations directed towards several health areas: controlling the risk factors, lifestyle (sleep, nutrition, and dietary habits), daily activities (physical activity, social participation, and relationship), and cognitive support and stimulation.

Our proposed procedure for managing the risk of mental and emotional long-term decline during and after SARS-CoV-2 infection is intended to cover a larger range of aspects that are associated with these cognitive disorders, starting with its design, comprising the management of the identified mental dysfunctionalities in conjunction with other co-morbidities of the elderly, and periodical assessment of the outcomes and feedback of the procedure, and last but not least, formulating potential proposals for improving medical practices, but also the political instruments that govern the field of medical and social care for the elderly.

**Figure 1:**
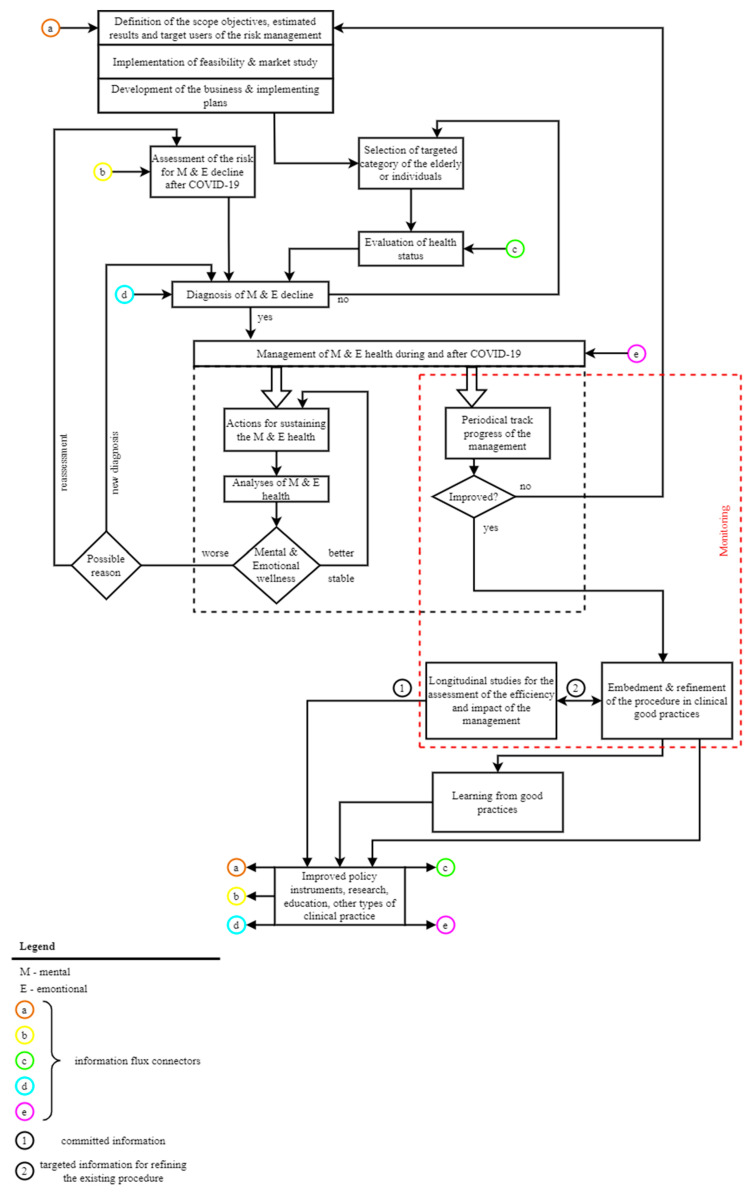
Diagram of the proposed procedure for managing the elderly’s risk of mental and emotional long-term decline during and after SARS-CoV-2 infection.

As is presented in Figure 1, the proposed procedure is designed to be scalable and able to evolve continuously according to the necessities at the individual or group level of elderly patients or, depending on the social/healthcare context, being provided with several steps that allow its improvement and upgrade.

The diagram presented in Figure 1 is detailed as follows:

The steps in designing the proposed procedure consist of clearly defining the *scope, objectives, estimated results, and target users of the risk management.* Briefly, it aims to better support the elderly with mental and emotional problems induced or aggravated by SARS-CoV-2 infection or COVID-19 pandemic.

It is supposed to become an efficient tool:

- To improve healthcare and social services provided by medical and social specialists;
- For better self-management of the health statuses of elderly patients, who are also assisted to be better aware and empowered regarding their own health.

Not less important is the estimated impact on improved policy instruments, research, education, or other types of clinical practice.

For a real-time response and a clear vision of the current situation of the local healthcare ecosystem (with a focus on mental and emotional issues), *implementation of feasibility and market study* should be performed, followed by *development of businesses and implementing plans*. In this phase, the results of national surveys—targeted for evaluating different issues related to the impact of COVID-19 pandemic on the elderly (as those presented in Section 2 and Section 3 of this paper)—are important for identifying the most relevant aspect and approaches. Thus, the risk management plan targeted for the mental and emotional long-term decline is designed, structured, adapted to local conditions, and developed in accordance with the real requirements of this category of vulnerable patients, but also those of medical and social service providers.

As it was stated previously*, the assessment of the risk for mental and emotional decline after SARS-CoV-2 infection* has crucial importance for managing the associated long-term decline. The results of related surveys, mental status tools for evaluating cognition (such as MMSE, MoCA, ADL, Free-Cog or Anxiety and fear of COVID scale—AMICO), or wearables/smart devices able to assess physiological changes through continuous remote monitoring are sources for generating a reliable identification of these risk factors. These factors should be concatenated with all the other risk factors used for evaluating the mental or emotional status of elderly patients, without necessarily being related to COVID-19.

Some of the above-mentioned risk factors are the same: age, gender, medical history, education, health status (including pre-existing mental and emotional disorders), way of living (with aspects related to loneliness, social relationship, and economic status), access to medical and social assistance, and access to digital technology, level of IT and health literacy, or ageism influences. Once identified, all these risk factors must be prioritized.

The *selection of targeted category of the elderly or individuals* aims to better implement risk management according to the main scope of the healthcare provider that applies this plan. This procedure can be used for improved management of mental and emotional disorders of a particular elderly patient or for a specific category of older patients, depending on the level at which it is applied and by whom it is applied (a physician, a medical unit or at the level of the health insurance system).

Once the targeted elderly patient(s) is/are selected, *an evaluation of their health status* is carried out with the help of traditional medicine, but also with the help of digital technology, including RMDSs.

The results of the evaluation of the health status corroborated the identified and prioritized risk factors are the basis for a reliable *diagnosis of mental and emotional decline*.

In this phase of the procedure, an evaluation of the current situation is performed. If no decline in mental and emotional status of the elderly patients that were previously infected with SARS-CoV-2 is identified, it returns to the step in which a patient or category of patients is selected.

If a decline is identified, the next phase of the procedure is activated, i.e., the management of mental and emotional health during and after COVID-19 pandemic. This one is structured in two parts: (1) appropriate healthcare assistance and services and (2) monitoring of the risk management plan.

(1) Appropriate healthcare assistance and services start with *actions for sustaining mental and emotional health*. Some of these most representative actions are:

- Implementing flexible coping strategies and programs to support the physical and psychological state of the elderly patients, their cognitive status, and physical activity;
- Increasing the participation, awareness, and empowerment of the elderly in the management of their mental, emotional, and health status;
- Supporting long-life learning in appropriate domains such as health or ICT for decreasing the digital divide, ageism influences, age-related dysfunctionalities, etc.;
- Enlarging the access to RMDSs;
- Improving the co-participative design and implementation of customizable remote health monitoring;
- Implementing preventative, proactive, and personalized protocols (including those supported by digital healthcare solutions) aiming to identify early the occurrence of a new risk factor or an abnormal mental/emotional state; act in response to a decline in mental and emotional state or crisis; and predict personalized patterns in the evolution of the elderly’s mental and emotional health;
- Ensuring the cognitive, behavioral, and rehabilitation training;
- Facilitating enhanced caretaker support that is personalized according to the elderly’s specificities;
- Creating a better framework for targeted mental and emotional screening.

These actions should be followed by *analyses of mental and emotional health*. These analyses imply:

- Checking the conformity of the actions with the current and updated legal framework;
- Correlating the cognitive and emotional distress with other health disorders, daily behavior, and specific issues associated with the COVID-19 pandemic framework;
- Performing correlational research aiming to detect the evolution in mental and emotional health before, during, and after COVID-19 crisis, in order to clearly identify the pandemic-associated disorders;
- Evaluating the role of RMDSs in the management of specific mental and emotional health.

The results of this phase of the procedure are evaluated in order to establish the state of mental and emotional wellness. If it is better or stable, the actions for sustaining mental and emotional health proved to be reliable, and they continue to be applied. If the mental and emotional wellness is worse, the possible reason is looked for:

- A new diagnosis is required or;
- A reassessment of the risk factors should be performed.

In both cases, the procedure is resumed accordingly.

(2) The Monitoring phase implies *periodical track of the progress of the management*. It is performed based on comprehensive questionnaires, surveys, and direct feedback for the implied users/actors. An assessment of the results is performed. If the results are not improved (i.e., the state of mental and emotional health of the elderly patients is not better or the healthcare resources have not become more efficient), it means that the whole risk management plan should be improved, so the procedure is restarted from the beginning in order to be refined or updated.

If the risk management proves to be improved, the next step consists of the *embedment and refinement of the proposed procedure in clinical good practices*.

In parallel*, longitudinal studies for the assessment of the efficiency and impact of risk management* must be performed. Between these two last phases of the procedure, real-time targeted information is shifted, shared, and used, aiming for the refinement of the procedure.

As the procedure is implemented in a medical unit, and in time it demonstrates its efficacy, reliability, and efficiency, it becomes a good clinical practice; the more good practices, the more new *learning from them* can be obtained.

All the committed information, data, and knowledge generated by the embedment and refinement of the proposed procedure in good clinical practices, longitudinal studies for the assessment of the efficiency and impact of risk management, and learning from good practices have great potential to sustain *improved policy instruments, research, education, and other types of clinical practice*. Additionally, starting from these improvements, the proposed procedure itself can be improved starting from different levels, as it is presented in Figure 1 in the areas where the information flux connectors are shown.

This approach sustains the scalability and flexibility of the proposed procedure.

### 2.3. Steps for Implementing Functionalities Provided by RMDSs in Accordance with the Proposed Procedure

Designing and implementing an RMDS targeted to support the management of mental and emotional disorders related to SARS-CoV-2 infection can be naturally integrated into the consecutive phases of the proposed procedure. Some of cases were already mentioned previously. Here are all the phases in the proposed procedure (presented in the diagram from Figure 1 in which the RMDSs can be used/implied:

- *Definition of scope, objectives, estimated results, and target users of the risk management*: the RMDS can be designed in a personalized manner, if possible, in a participative way with the direct involvement of the elderly patients and medical specialists;
- *Assessment of the risk for mental and emotional decline after SARS-CoV-2 infection*: some of the elements on which the assessment is based comprise data and information obtained from remote monitoring performed through RMDSs, as well as the insight obtained from different surveys, statistics, or relevant open data sources with the help of Artificial Intelligence or Big Data Analytics that can be included as capabilities in RMDSs;
- *Selection of targeted category of the elderly or individuals*: they can be selected via different questionnaires included in RMDSs;
- *Evaluation of health status:* gathering health and lifestyle data based on the primary functionalities of RMDSs is completed with their data processing support functionalities;
- *Diagnosis of mental and emotional decline*: predictive models, personalized differently depending on the elderly individual patient or category, mental or emotional disorders, social environment, etc., are very powerful tools provided by RMDSs and able to support the diagnosis and medical decision making;
- *Management of the mental and emotional health during and after COVID-19 pandemic:* all the functionalities provided by RMDSs (gathering health and lifestyle-associated data, data analytics, predictive models, decision-making and informational support, long-life learning, etc.) are compulsory nowadays for sustaining and analyzing the health status, in our case, the mental and emotional ones;
- *Monitoring of the risk management plan*—namely, the *Periodical track progress of the management* and *the Longitudinal studies for the assessment of the efficiency and impact of the management* can be sustained by specific functionalities provided by RMDSs, such as targeted questionnaires or statistics based on the users’ feedback, medical outcomes, and financial and human resources involved in the medical care given to the elderly due to mental and emotional disorders associated with COVID-19.

In conclusion, for a successful implementation of dedicated RMDSs for sustaining long-term personalized management of mental and emotional decline, their targeted users should be involved throughout the entire development cycle, from the definition of technical and (non)functional requirements and specifications, to testing in laboratory and real conditions and, in the longer term, to the upgrades required by the dynamism of the field of smart devices and emerging technologies. No less important is the need for the RMDS architecture to be scalable, flexible, secure, reusable, agile, robust, and age-friendly in order to be easily adapted and integrated into different clinical environments or elderly’s homes.

## 3. Results

### 3.1. Results from a Sample of Romanian Surveys on COVID-19

(a) Results from IRES SURVEY “A month of loneliness” [59]

**Figure 2:**
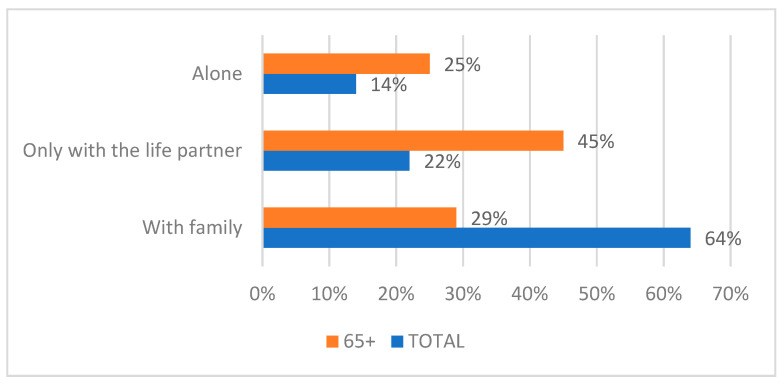
How did you get through most of the first 30 days of the pandemic?

**Figure 3:**
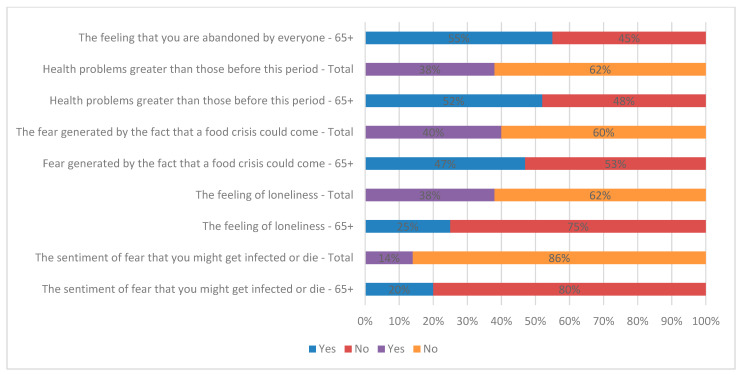
Since declaring the state of emergency, have you felt any of the following states/feelings?

The collected data proved that the elderly (aged 65+) are the ones who, during this period, also face greater health problems than before the COVID-19 pandemic, experiencing, in high proportions, feelings of loneliness and fear of death caused by SARS-CoV-2 infection, but also the fear of a food crisis (see Figure 2 and Figure 3).

It can be observed from Figure 2 that the fear of infection or death caused by SARS-CoV-2 infection, the feeling of loneliness, the fear of a food crisis, and the existence of bigger health problems have higher percentages in the case of the elderly than in the rest of the entire population. Such problems have a strong impact on their psycho-emotional state.

(b) Results from IRES SURVEY “Romanians after 2 years of COVID-19” [60]

According to the research, one in four people aged 65+ in the urban environment, that is, over 450,000 people, face a high degree of loneliness, and 36% of respondents feel an average degree of loneliness.

Factors such as poor health status or the loss of a life partner contribute to restricting social interactions, such that 28% of the elderly end up socializing with a maximum of four people in a whole month.

Oppressive loneliness affects the elderly, including their health: thus, one in four seniors declare that they have poor physical and mental health. Among people with a high degree of loneliness, 39% have physical problems. Health problems cause one to spend more time indoors: 64% of seniors spend most of their time performing household activities such as cooking or cleaning, occupations used as mechanisms to combat loneliness.

Their routine looks like this: 43% go for a walk exclusively around the house or in the park, 33% watch TV, and only 27% have activities outside the home, including gardening or other hobbies.

Those who experience a high degree of loneliness leave the house even less (18%).

(c) Results from KANTAR ROMANIA, at the request of the Never Alone—Friends of the Elderly Association [61]

In the context of the possibility that the intensity of the current pandemic will decrease in the next period, the survey wanted to evaluate, at the same time, the degree of concern regarding the current pandemic, but also the possibility of a new pandemic, as well as the time horizon in which Romanians expect the current pandemic to end.

The results show that although the COVID-19 pandemic had a significant impact on the health and psycho-emotional state of Romanians, 21% of older Romanians are no longer worried at all about the current pandemic. At the moment, 58% are worried about a war in the region.

More than half (53%) of the elderly participants in the IRES study believe that the current pandemic will end this year. At the same time, however, one in three older people in Romania (36%) believe that the emergence of a new pandemic in the near future is very likely.

The results regarding the degree of concern of the 65+ population relative to the COVID-19 pandemic are presented in Table 4.

Regarding the good changes brought about by the COVID-19 pandemic among the elderly, they are mainly related to the time available to be spent with the family and to the way of protecting their health more during the pandemic.

Regarding the bad changes brought about by the COVID-19 pandemic among the elderly, they are mainly related to the restrictions and limitations of movement, the limitations imposed on social life and interaction with others, the limitation of access to medical services, the alteration of the state of health, the states of stress related to the fear of not contacting the virus and the pain related to the death of a close person.

The elderly in Romania believe that they have learned something new thanks to the pandemic, something that they would not have learned in another context. Along with the functional elements related to respecting the hygiene rules and maintaining social distance or those related to health or saving money, many aspects aimed at empathy and the relationship with other people are also included.

Freedom of movement is the first aspect that Romanian seniors have in mind when they are asked what would be the first thing they would do when all the restrictions in Romania are lifted.

### 3.2. Assistance of the Elderly through Dedicated Functionalities of RO-SmartAgeing System

#### 3.2.1. Brief Presentation of RO-SmartAgeing System

As the isolation and apprehension brought on by the pandemic have strongly influenced the mental health and wellbeing of the elderly, even though it is still an uncertain situation to visit them in person, it is critical to keep a regular connection in order to look after any changes or signs that could lead to a senior’s mental or behavioral health concern. In this context, an RMDS for sustaining the mental health of the elderly in their home environment is an enhanced necessity.

RO-SmartAgeing is a system designed to offer personalized in-home services for an elderly person, based on the remote monitoring of various health and ambient parameters and daily activities, across a range of preventative and proactive features targeted to sustain a healthy, independent, and active life; specific requirements for the elderly in order to avoid unexpected concerns related to their mental or behavioral problems.

The smart environment encompassed into the system gathers a set of devices that can be customized and tailored according to elderly patients’ health and needs. Their most important technical characteristics are presented as follows:

- Withings MoveECG smartwatch [65]: With a diameter of 38 mm and a weight of 32 g, it can be used to track health parameters (Electrocardiogram—ECG sensor) as well as daily activity information (altimeter and accelerometer sensors). Based on Bluetooth Low Energy (BLE) syncing with a smartphone, it is considered a smartwatch for monitoring day and night activity;
- Withings Sleep Analyzer [66]: Real-time monitoring of sleep-related health data is necessary in order to have an overview of the sleep patterns of the person. It is an easy-to-use device as it is placed under the bed mattress, and it is configured with two sensors: a pneumatic sensor (monitors the body movements across the mattress, cardiac rhythm through ballistocardiography, and respiration rate) and a sound sensor (detects audio signals associated with snoring and any discontinuances in the breathing episodes);
- Withings Thermo [67]: With 16 integrated infrared sensors, this device has a weight of 75 g and a temperature range of 35 °C–43.2 °C (and a clinical accuracy of ±0.2 °C). It is a no-contact device that provides an ultra-hygienic measurement, and the data are automatically synchronized via Wi-Fi into the app;
- Withings Body+ Smart Scale [68]: A full body composition analysis, based on four weight sensors and a body position detector, can be performed using this device. It uses bioelectrical impedance technology to deliver a low-amplitude electrical current through the user’s body and measure additional biological tissue resistance;
- Withings Blood Pressure Monitor (BPM Core) [69]: A wireless blood pressure monitor (BPM) is necessary for constantly monitoring systolic and diastolic blood pressure for the elderly. This device has a digital stethoscope and three stainless steel electrodes for monitoring not only blood pressure, but also the ECG and valvular sounds;
- Gait band: It can detect the acceleration of the user’s body changes and the speed of the body rotation based on the integrated 6-axis accelerometer and gyroscope sensor that has a 5 V input voltage and an I2C interface which makes it easy to program and configure after the user’s specificities.

The HealthMate application collects all the data from the Withings-related devices, and the RO-SmartAgeing system is configured in order to transmit the information into the Cloud database for additional analysis and processing.

**Figure 4:**
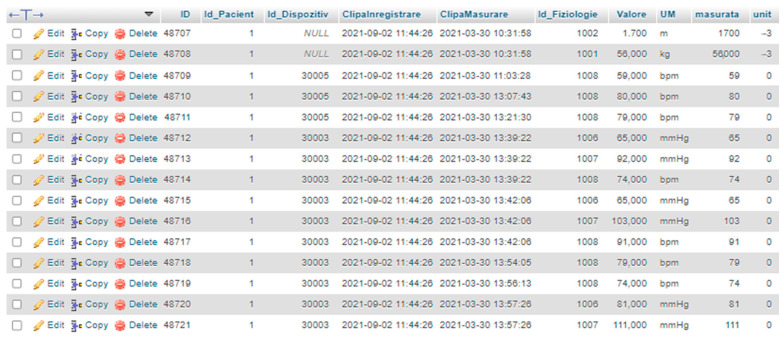
ROSmartAgeing Cloud database illustration of a series of consecutive measurements of multiple parameters performed with the devices.

The gait band is also programmed to send the relevant data into the Cloud database. The RO-SmartAgeing Cloud database is integrated into the ICIPRO Cloud platform [70], and it is configured to collect a wide range of data (see Figure 4).

**Figure 5:**
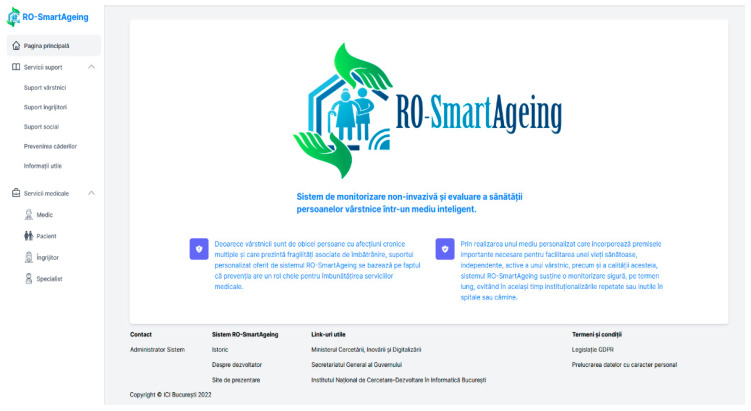
The main page of the RO-SmartAgeing platform (in Romanian).

After the data are gathered into the Cloud database, they can be further visualized in the RO-SmartAgeing platform. The RO-SmartAgeing platform has five types of users: the physician, patient, caregiver, specialist physician, and administrator. Each type of user can access a module corresponding to his role and needs to authenticate in order to visualize/edit the information. The main page of the platform is presented in Figure 5, and it contains both the support services component and the medical component, which are described in the next subchapter.

In the above figure, the left side of the main page describes the menu of the platform: the button towards the Home page, the “Support Services” button (with the following sub-menu options: “Elderly support”, ”Caretakers support”, “Social support”, “Fall prevention“ and “Useful information”) and the “Medical Services” button (with the following sub-menu options: “Physician”, “Patient”, “Caretaker”, “Specialist physician” categories for specific authentication). In the center, there is the logo of RO-SmartAgeing, as well as a description of the system. Other relevant links and information can be found in the footer area of this page.

#### 3.2.2. Specific Capabilities of RO-SmartAgeing System

Various factors can lead to a significant increase in the risk of developing mental health problems. However, a preventative approach through maintaining a good quality of life for one individual can have an enormous impact on his/her behavior and mental state over time. The remote monitoring of a person who is prone to mental decline is a serious aspect that needs to be taken into consideration when it comes to diminishing the risk of emotional and mental long-term decline after a SARS-CoV-2 infection.

The RO-SmartAgeing system is provided with specific capabilities for addressing improved preventative and proactive support for diminishing the risk of emotional and mental long-term decline after SARS-CoV-2 infection.

RO-SmartAgeing has a significant impact through the novel integrated solutions regarding monitoring, wellbeing, and support services enabling an enhanced patient quality of life, especially for the elderly who need to improve and sustain not only their medical health but also their mental health, wellbeing, and behavioral health, as well as their social skills.

The RO-SmartAgeing system encompasses the medical and support service components. The medical component aims to incorporate in a single point, with controlled access, a series of functionalities adapted to each type of user (physician, elderly person, caregiver/family member, specialist physician). It is designed to support personalized monitoring and management of a senior’s health in a friendly environment. In addition to this, the support services component is focused on providing specific information and recommendations related to the daily life needs of the elderly: information on aging-related conditions and diseases and recommendations for healthy, independent, social living.

Monitoring mental illness symptoms is very important because they can influence emotions, thoughts, or behaviors among the elderly; there are sensors, wearable devices (RO-SmartAgeing smart environment), questionnaires, and cognitive tests encompassed into the RO-SmartAgeing system that could track and sustain the quality of life of the patients.

- **The Medical Component**

RO-SmartAgeing is a solution that could help in treating and understanding the needs of elderly patients with mental illness or behavioral difficulties. The medical component gathers several Withings devices: the MoveECG smartwatch, Sleep Analyzer device, thermometer, Smart Scale or BPM, as well as integrating a gait band for fall detection and prevention, together with a memo for current activities and a messages service for maintaining strong communication between the elderly, physician, family members and other medical specialists. *The RO-SmartAgeing smart devices* and their capabilities associated with mental or behavioral aspects are described below:

 a.  *MoveECG smartwatch*: Electrocardiogram (ECG) monitoring and heart rate

The human brain is the most important part of the nervous system. Thus, a reduced heart rate variation is correlated to stress or mental health issues, resulting in loneliness, anxiety, and self-criticism [71,72]. At the same time, an increased heart rate variation implies a solid ability to tolerate stress. However, a high heart rate variation is prone to heart disease. The MoveECG smartwatch is developed to continuously monitor the heart rate, providing real-time data streaming. The smartwatch can be easily placed on the patient’s wrist, allowing the continuous transmission of the heart rate and accurately monitoring the ECG when it is needed. This allows healthcare providers to interfere in any emergency related to significant alterations in the patient’s heart rate. Meanwhile, measuring the ECG can be of extreme help in order to determine if a patient suffers from depression or bipolar disorder [73].

 b.  *Withings Sleep Analyzer: Sleep tracker*

The connection between sleep and mental health is considered bi-directional, as low quality of sleep can lead to poor mental health and vice versa [74]. Early waking in the morning, augmented by low energy, distraction, sadness, or loss of appetite, may suggest symptoms related to depression. On the other hand, an incapacity of sleeping or a major decrease in sleep associated with a high level of activity implies several signs of maniacal behavior. Anxiety is also related to sleep problems, represented by awakening, increased sleep onset latency, and low quality of sleep. Sleep deprivation is also related to obsessive–compulsive disorder, as well as detecting sudden awakening through panic episodes during sleep may indicate symptoms of panic disorder. In this context, the Withings Sleep Analyzer can detect any signs related to the movements, sleep cycles, and sleep score (duration, recovery, the number of interruptions, regularity, or time to sleep). The installation of the Sleep Analyzer is firstly based on placing it entirely under the mattress in order to be positioned underneath the chest of the sleeping person. After plugging it into an outlet using the provided adapter, it starts to measure the relevant parameters, making the proper evaluation the next morning. The heart rate is also measured using the Withings Sleep Analyzer as it is an essential parameter to be monitored while sleeping, as it indicates the time covered in the deep sleep phase as well as keeps good management of the heart health.

 c.  Withings Thermo: body *temperature*

Temperature is another parameter relevant to monitoring the mental health of the elderly. Studies [75] have shown that stress can induce an increase in body temperature. Psychogenic fever can be induced by any kind of stress, a continuous fear of COVID-19 being one cause of social stressors that can eventually trigger a febrile response, significantly impacting the health of the elderly. The Withings thermometer is capable of accurately measuring body temperature and sending the data immediately to the RO-SmartAgeing dashboard. The thermometer is easy to use as it only requires a slide over the patient’s forehead. It measures the temperature at the temporal level, considering that the temporal artery is considered the best place to detect any changes in the body temperature. If there are any significant variations being measured (a temperature higher than 37.5 °C), an alarm is triggered in order to alert the healthcare specialists.

 d.  *Chest band: accelerations and position*

Mobility is linked to the symptoms related to schizophrenia or other neurodegenerative disorders. The risk of falling is also enhanced by mental health issues, but the lack of mobility worsens health problems, reducing social interactions and leading to depression or other mental or behavioral conditions [76]. The RO-SmartAgeing chest band proposes a smart device that could track the position of the elderly. The chest band is placed on the patient’s body at the chest level, in order to detect imbalances, movements, positions, and eventual falls, based on accelerations. It can also detect any changes in posture, identifying early the possibility of falling, triggering an instant alarm on the RO-SmartAgeing dashboard. In this sense, the chest band can sustain a social life for the elderly, helping them toward continuous monitoring of eventual falls or movements.

 e.  *Withings Body+ Smart Scale*: weight and other body composition parameters

Changes in eating habits directly influence weight loss/gain, which can often be correlated with depression [77]. A continuous decrease in weight is interpreted as a diagnostic for depression; depressive people not having a normal appetite due to the lack of pleasure from eating. On the other hand, an increase in weight can be another aspect of depression as the elderly are more likely to not engage in physical activities because of tiredness or age-related aspects even though they consume a large amount of food for their own comfort. In this situation, there is the need to continuously monitor the body composition parameters in order to track any substantial change in weight loss/gain. The Withings Body+ Smart Scale can monitor a wide range of parameters related to the human body composition and transmit the data through the RO-SmartAgeing dashboard to be seen by the health providers in order to take necessary actions: change the patient’s environment, current activities memo or treatment plan according to the measured values and diagnostic.

 f.  *BPM Core:* systolic and diastolic blood pressure

Anxiety associated with the COVID-19 pandemic has proven to have symptoms in blood pressure. Several studies [78,79] have shown an increase in the value of home monitoring systolic blood pressure among the elderly, leading to a much greater risk of cardiovascular issues. Thus, RO-SmartAgeing can help in preventing anxiety aspects in older adults through the easy use of BPM Core. The BPM is placed on the patient’s arm and can wirelessly transmit the data to the RO-SmartAgeing system, allowing the healthcare providers to see the home-monitoring of blood pressure variation values over a certain period to detect any significant changes that can occur at the mental level of the patient.

In addition to the smart devices capable of gathering health data for the evaluation and monitoring of mental, behavioral, or emotional features related to the elderly in the context of the COVID-19 pandemic, there are two functionalities of the RO-SmartAgeing system that can improve an elderly patient’s quality of life, through *monitoring the current activities* and maintaining a social life by keeping up with the other related users through *messages*.

- *Current activities memo functionality*

In the context in which an elderly patient is prone to problems related to cognitive degeneration and mental imbalances, monitoring the current activities can provide support for maintaining a good social life and self-care, and improve the quality of life.

Generating alerts in case of non-compliance/omission of a recorded activity implies sending reminders for administering pills or respecting the provided treatment. The RO-SmartAgeing functionality of the memo for current activities allows personalized recommendations for the patient’s care in terms of sustaining their activities in order to maintain efficient healthcare and good quality of life, as mental and physical health are essential in pursuing a healthful lifestyle to reduce any risks of diseases. Keeping and maintaining an organized schedule for activities can improve the endurance of a person in terms of cardiological functionality, respiratory system, muscular strength, or body composition.

In addition to this, mental health is also sustained through physical activities, having a great impact on boosting the patient’s emotional, social, and psychological wellbeing [80].

- *Messages functionality*

Keeping efficient communication between the patient and the physician is mandatory in terms of the post-COVID-19 pandemic, as there is the need to remotely correspond with medical professionals, caregivers, or other medical specialists when any healthcare-related problems may appear. For example, the caregiver, as an authorized user of the RO-SmartAgeing system and being responsible for the elderly person, can send a message, in real time, to the physician if there are any issues regarding the health or the wellbeing of the patient. Once any messages are received by the physician, any further clinical investigations can be based on the measured health data or on the activities the patient is doing.

- **The Support Services Component**

The support services component aims to provide both seniors and caregivers with information and recommendations specific to the daily life needs of the seniors. It attempts to provide the necessary information specific to the older people’s conditions and recommendations for a healthy, independent, and social life, and it also allows the possibility of assessing the mental and behavioral capabilities of the elderly using cognitive tests.

  *A.* *Social relationship support and cognitive abilities module*

This module offers the opportunity for the elderly and their caregivers to assess the patient’s cognitive abilities and improve their social relationships through interactive tests and relevant information regarding the social aspect of their life. Social networks have a great impact on the quality of life of the elderly, especially in the emotional quality of social relationships. They offer older people the opportunity to renew or develop social contacts and to actively engage in their own community, thus preventing the social isolation of older people and the feeling of loneliness that occurs in the case of retirement, health deterioration, or COVID-19-related quarantine.

The *Social relationship support and cognitive abilities* module covers two sections: (self-)assessment cognitive tests and social support for the elderly.

The purpose of the first section is to allow elderly people to self-assess their cognitive abilities or let their caregivers help them in their evaluation through the provided tests in the RO-SmartAgeing application. Evaluating cognitive abilities or neurological impairments is essential for the elderly, especially in the COVID-19 era, as the mental state or condition can deteriorate.

Among the smart devices encompassed in the smart environment, RO-SmartAgeing has the capability of providing a series of cognitive tests for the patient in order to be evaluated by themselves, or by the caregiver or physician. The cognitive tests integrated into RO-SmartAgeing are either

- developed by medical specialists that use the RO-SmartAgeing system as support in the provision of specialized medical services;

or

**Figure 6:**
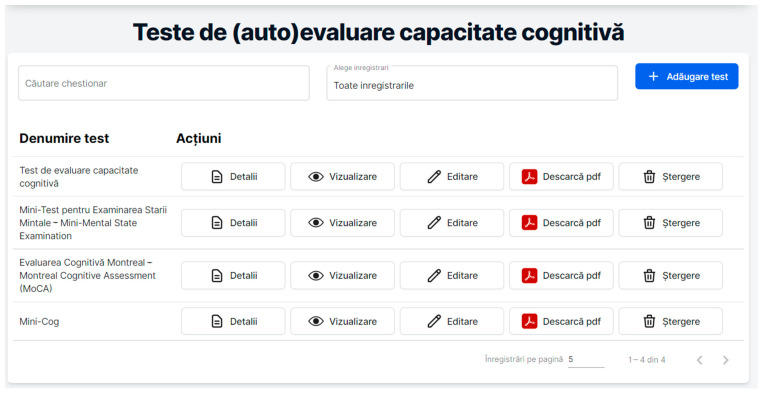
RO-SmartAgeing (Self-)assessment cognitive tests (in Romanian).

- based on some of the most popular screening tools for cognitive impairments, such as *MMSE* [22], *MoCA* [23], and *Mini-Cog* [29] (see Figure 6).

Figure 6 describes the (Self-)assessment cognitive tests (Teste de (auto)evaluare capaciate cognitive) page in the RO-SmartAgeing platform. There are two boxes under the page title for the “Search test (Căutare chestionar)” functionality (the users may search, based on certain keywords, the kind of test that they are looking for) and the “Choose recording (Alege înregistrări)” functionality (the users can select “All the recordings (Toate înregistrările)” option which displays all the tests from the database or choose other options for displaying associated information). Next to these boxes, there is the “Add test (Adăugare test)” button which enables the authorized user to add another test. Below, under the title ”Test name (Denumire test)”, there is a list of four current available tests: a cognitive assessment test uploaded by one physician (Test de evaluare capacitate cognitive), and the MMSE, MoCA, and Mini-Cog evaluation tests. The ”Actions (Acțiuni)” that can be applied to the tests are the following: “Details (Detalii)” (for showing the details of a certain test), “View (Vizualizare)” (for visualizing the test), “Edit (Editare)” (if the physician is authenticated, the “Edit” button is available in order to modify or add information), “Download (Descarcă pdf)” (if the patient wants to download and print the test) and the “Delete (Ștergere)” option (for deleting the test).

The MMSE is easily downloadable from the RO-SmartAgeing platform. It is administered with the assistance of the caregiver in order to assure the robustness of the answers, and via telehealth, it supports the patients for whom distance, expenses, or mobility are considered obstacles to proceeding with face-to-face in-hospital visits. Thus, the elderly patient who is monitored using the RO-SmartAgeing system can be periodically evaluated in terms of the following cognitive functions: time and space orientation, enrollment, awareness and calculation, recall, praxis, and language.

RO-SmartAgeing also provides the possibility of downloading the MoCA test. The test is performed by the elderly patient assisted by the caretaker and, optionally, via videoconference, by the physician. The results of the MoCA test can provide information in addition to the evaluations of the mental state of the patient obtained following the use of other tests accessible through the RO-SmarAgeing system, such as the level of orientation, short-term memory, visuospatial capacity, abstraction, attention, or use of language.

The Mini-Cog test can be easily downloaded from the RO-SmartAgeing platform. As the elderly patient (assisted by the caretaker) has only to repeat three words and draw a clock, this test can be easily performed and interpreted in a step-wise fashion. It provides a neuropsychological evaluation of the patient, which contributes in a reliable way to the early diagnosis of some cognitive disorders.

**Figure 7:**
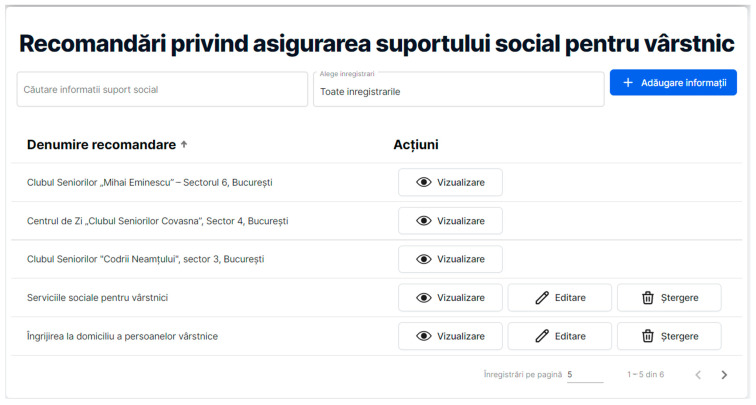
Recommendations regarding the provision of social support for the elderly (in Romanian).

The second section of the *Social relationship support and cognitive abilities* module aims to synthesize the benefits brought to the elderly by ensuring quality social support (see Figure 7), and thus the advantages brought by the social involvement of the elderly in the community, the facilitation of new social contacts, as well as the benefits of intergenerational activities are provided.

The presented information aims to offer the necessary support for the development of social relationships (with physicians, relatives, friends, and people with similar concerns), such as the elderly’s access to support groups.

In the above figure, the page entitled “Recommendations regarding the provision of social support for the elderly (Recomandări privind asigurarea suportului social pentru vârstnic)” enables the user to search for certain information on the webpage, but also to select data based on the “Search information (Căutare informații suport social)” box and “Select recording (Alege înregistrări)” box, respectively. The “Add information (Adăugare informații)” button allows any authorized user to introduce relevant information. The recommendations for social support are displayed under the title ”Recommendation name (Denumire recomandare)”, containing relevant information about ideas for establishing social interactions among the elderly (for example, information about various seniors’ clubs (Clubul Seniorilor ”Mihai Eminesccu” – Sectorul 6, București), day centers for old persons (Centrul de zi ”Clubul Seniorilor Covasna”, Sector 4, București), etc.). Some Actions (Acțiuni) are available for the authorized user. All the recommendations are associated with the “View (Vizualizare)” button that enables the display of the accessed data. Although all the information stored in the RO-SmartAgeing platform can be modified only by the authorized health specialist that introduced it, some recommendations can be edited (the “Edit (Editare)” button) or erased (the “Delete (Ștergere)” button) only by the webpage administrator.

  *B.* *Support services for elderly people*

This category of services will provide informative and interactive support for the conditions and problems faced by the elderly to help them strengthen a healthy and independent life. The subcomponent will allow the elderly to self-assess their quality of life using interactive questionnaires.

The quality of life of the elderly includes both subjective elements (psychological wellbeing, autonomy, the activity carried out pursuing a certain goal, social relationships, spirituality, and identity) and objective elements (physical and care environment, physical and mental health, level of functioning, and socio-economic status).

The *Support services for elderly people* module covers three sections: information on aging-related conditions, healthy and independent living information, and quality of life and wellbeing self-assessment questionnaires.

**Figure 8:**
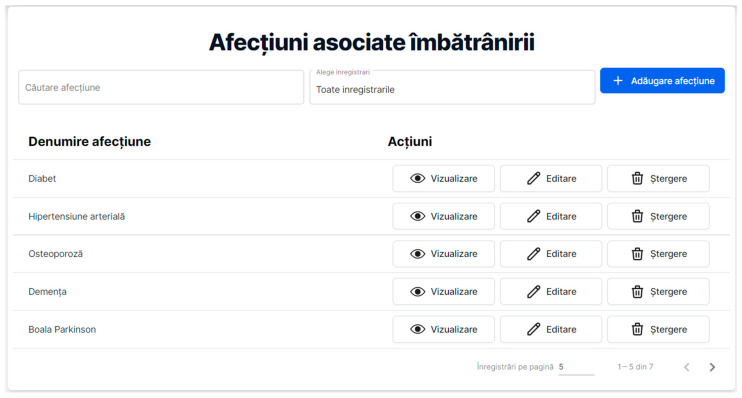
Information on aging-related conditions section (in Romanian).

The purpose of the first section is to provide anyone interested with basic information about the most important conditions, as well as their specificities, associated with aging. It is important that the specifics of a geriatric condition be understood by non-medical, elderly, or other interested people (see Figure 8).

In the above figure, the “Ageing-related health conditions (Afecțiuni associate îmbătrânirii)” webpage displays the “Search condition (Căutare afecțiune)” and “Select recording (Alege înregistrări)” boxes, as well as the “Add health condition (Adăugare afecțiune)” button for authorized users. Below, under the title ”Health condition name (Denumire afecțiune)”, a list of such health conditions from the RO-SmartAgeing database is displayed: diabetes (Diabet), hypertension (Hipertensiune arterială), osteoporosis (Osteoporoză), dementia (Demență), and Parkinson’s disease (Boala Parkinson). On the right, there are the actions available for these items: the “View (Vizualizare)” button for displaying the stored information, and the “Edit (Editare)” and “Delete (Ștergere)” buttons available for an authenticated user in order to modify that record.

The displayed centralized information on the main conditions associated with aging can be accessed by anyone without the need to be registered. It is the authorized user (physician or healthcare provider) who will enter this structured information in a way that is as accessible as possible to the elderly. In this context, this can increase the level of awareness of the risks associated with aging and offer support for better management of one’s own health condition in order to sustain his/her mental health.

The second section, healthy and independent living information, aims to gather all the relevant information for elderly users and their caregivers in order to support the process of active and independent aging through a series of preventive measures and recommendations toward monitoring their health status.

The information is mostly related to chronic conditions and premature aging, considered risk factors for mental and behavioral health degeneration: alcohol and tobacco consumption, unhealthy diets, and the inclusion of physical exercises in daily activities. In addition to this, cognitive health—the ability to think, learn and retain clearly—is an important component of carrying out everyday activities.

**Figure 9:**
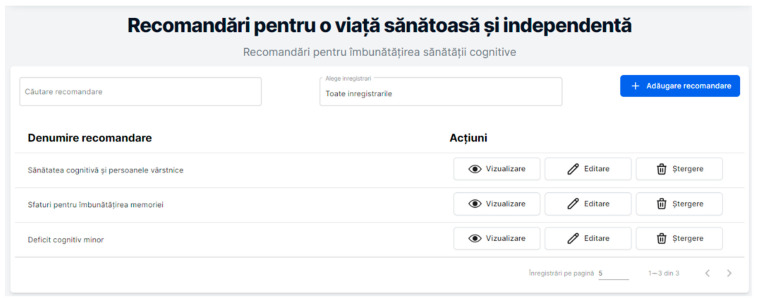
Healthy and independent living information section (in Romanian).

Cognitive health is only one aspect of overall brain health. Brain health can be affected by age-related brain changes, injuries such as stroke or traumatic brain injury, mood disorders such as depression, substance use disorder (drug addiction), and diseases such as Alzheimer’s disease. While there are some factors that affect brain health that cannot be changed, there are many lifestyle aspects that could make a difference. This section presents information and recommendations to improve the cognitive ability of the elderly (see Figure 9).

Figure 9 displays the “Recommendations for living a healthy and independent life (Recomandări pentru o viață sănătoasă și independentă)” module. The user has access to the boxes for “Search recommendation (Căutare recomandare)” and “Select recording (Alege înregistrare)” functionalities that are useful in the case of a list with a lot of recordings from the database. The button for “Add a recommendation (Adăugare recomandare)” allows the authenticated user to upload the related information into the RO-SmartAgeing database. The displayed list under the title ”Recommendation name (Denumire recomandare)”, contains three recommendations: “Cognitive health and the elderly (Sănătatea cognitivă și persoanele vârstnice)”, “Advices for improving memory (Sfaturi pentru îmbunătățirea memoriei)”, and “Minor cognitive deficit (Deficit cognitive minor)”. As previously mentioned, the available buttons for these recommendations are as follows: “View (Vizualizare)” to visualize the stored information, “Edit (Editare)” and “Delete (Ștergere)” to modify the information by the authorized users.

The last module, Quality of life and wellbeing self-assessment questionnaires, provides anyone interested with relevant questionnaires to self-evaluate the level of their quality of life and overall wellbeing. This module offers the possibility to increase the awareness of the elderly concerning the risks associated with aging, and as a result, it helps them in making decisions about the management of their own health.

The questionnaires in this module refer to physical and psychological health, social relationships, and the elderly person’s home environment.

## 4. Discussion

The impact of the pandemic on the psycho-emotional state of seniors due to poor physical or mental health and accentuated by several risk factors that generated anxiety, loneliness, depression, fear of health problems, infection, or even death, was mentioned as an outcome of some surveys undertaken in Romania during or after the COVID-19 pandemic. The elderly in Romania say that during this period, they felt anxious and agitated to a greater extent than before the pandemic, they felt sad, depressed, or hopeless more than before the pandemic, and they felt that they could not stop worrying or keep their worries under control.

Such a result constitutes the basis of the participatory design of new RDMSs. The elderly have to be actively involved in the design process to help ensure that the result meets their needs and is usable even during pandemic periods.

Our proposed procedure for managing the elderly’s risk of mental and emotional long-term decline after SARS-CoV-2 infection *creates a framework* for the development of personalized, proactive models specific to certain cognitive conditions, aiming to perform the following:

- Prevent the deterioration of the elderly’s health or incidents with serious consequences;
- Support and prolong an independent, active, and dignified life for elderly patients;
- Recover in a familiar environment after post-traumatic incidents associated with the COVID-19 pandemic;
- Facilitate ways to migrate the medical system towards personalized and accessible healthcare centered on the elderly and their associated mental and emotional disorders;
- Update the way of interfacing with the elderly and the provision of medical services based on new and innovative digital technologies;
- Improve good clinical practices and their broader implementation, as well as the legal framework associated with the medical consequences of the COVID-19 pandemic over the elderly population.

Among the most representative *barriers* to a successful implementation of the proposed procedure for managing the elderly patient’s risk of mental and emotional long-term decline after SARS-CoV-2 infections are the following:

- Digital divide and digital literacy mainly for the elderly, but also for some of the healthcare specialists;
- Ethical issues;
- Physical limitations of the patients;
- Cognitive frailty of the patient;
- Lack of motivation and the existence of feelings of unfair care perceived by the elderly;
- Lack of a fundamental framework for longitudinal studies, considering the relatively short time since the beginning of the COVID-19 pandemic;
- Ensuring compliance with government guidance;
- An unbalanced multidisciplinary approach to the mental and emotional disorders of the elderly.

The efficient management of the risk of mental and emotional long-term decline after SARS-CoV-2 infection is definitively based on the deep implication of the elderly patient and their attending physicians.

As the COVID-19 pandemic may impact not only the elderly’s health, but also their mental health and wellbeing, remote health monitoring is necessary to assess and keep them updated with their vital signs and key information regarding social support, their cognitive state, or recommendations for a healthy and independent life.

In order to be proactive, preventative, personalized, and participative, a supporting RMDS must rely on their demands and necessities from the design stage. A participative approach allows the specification of the most appropriate technical and (non-)functional criteria and the identification of a large range of risks (for instance, the risk of triggering false alerts or ignoring age-friendly functionalities).

The RO-SmartAgeing system is an RMDS in which the patient design replaced the patient centricity and in which the elderly patients with potential progressive cognitive impairments are engaged in advanced personalized care models.

RO-SmartAgeing incorporates the development and validation of a system for monitoring and evaluating the health status of the elderly that integrates non-invasive, wearable physiological (Withings MoveECG smartwatch, Withings Sleep Analyzer, Withings Thermo, Withings Body+ Smart Scale, and Withings Blood Pressure Monitor) devices and movement sensors (gait band) to collect medical parameters, to monitor daily activities and lifestyle of the person, all integrated into a smart environment as well as a cloud platform for data storage and aggregation. An added value brought by RO-SmartAgeing is the possibility of (self-)assessing the mental and emotional health of the elderly, considering the vulnerability of their mental state in terms of the COVID-19 pandemic. This assessment is corroborated by physical evaluations, lifestyle, daily activities, and medical history, and thus, a broad range of comprehensive information is established and prepared for further analytics and supporting healthcare functionalities.

Monitoring a wide range of health parameters and offering constant and necessary information regarding the daily life, medical and social needs of the elderly, RO-SmartAgeing is a challenging solution for sustaining mental and behavioral health in times of COVID-19, especially in Romania.

The main functionalities of the RO-SmartAgeing system that can reliably support the management of the elderly’s risk of emotional and mental long-term decline after SARS-CoV-2 infection are as follows:

- Centralization of information about health and lifestyle (previous and current);
- Lifestyle monitoring;
- Personalized monitoring of biomedical, environmental, and movement parameters;
- Remote assessment and diagnostics;
- Assistance for elderly patient’s autonomy and emotional and mental wellness at home;
- Alerting in case of a physical accident, mental disorder, or in case of detection of an unusual emotional situation;
- Establishing, maintaining, and improving social relationships;
- Support for people who care for the elderly.

Based on the stored data, additional functionalities are implemented that allow the following support services:

- A treatment plan;
- A reminder of the current activity;
- A diagnosis.

The RO-SmartAgeing system has the following main features:

- It is preventive, proactive, and customizable according to the specificities of the elderly, but also according to the evolution of their health condition;
- It supports an integrated and participative management of the health status of the elderly;
- Through its functionalities, it sustains active, independent, and healthy aging, including responsibility, empowerment, and direct involvement of the elderly in managing their health and lifestyle;
- It provides security and confidentiality of medical and personal data;
- It is scalable and flexible.

**Limitations**: As the RO-SmartAgeing pilot system has just been completed and since it has been tested only in a laboratory environment, only some of the steps from the proposed procedure for managing the elderly’s risk of emotional and long-term decline after SARS-CoV-2 infection could be implemented.

**Future work**: The RO-SmartAgeing system will be implemented in a clinical environment, as it is already discussed with some legal entities and physicians from private and public clinics. In this respect, some preliminary stipulations and action plans have been initiated with the National Institute of Gerontology and Geriatrics “Ana Aslan” and Hospice Care for the Elderly of the Bucharest Municipality “Academician Nicolae Cajal”. After a representative number of elderly patients (the target number for the beginning is 100) are enrolled in a legally established trial, we estimate that in three months after the beginning of the monitoring and management of the mental and emotional health of an elderly person with the support of the RO-SmartAgeing system, the first periodical track progress of the management can be initiated. The appropriate periodicity for this tracking will be established according to the physicians’ demands and evaluations. The phase for embedment and refinement of the procedure in good clinical practices is estimated to first start after another three months.

## 5. Conclusions

The COVID-19 pandemic has had a significant impact on the elderly, with potentially dramatic long-term repercussions on their mental and emotional health. The impact of the pandemic on the psycho-emotional state of Romanian seniors is due to the lack of predictability, the avalanche of information, often contradictory, and the imposition of restrictions that some people have never faced during their lives, were elements that generated, in the two years, states of fear, anxiety or stress. Since the spring of 2020, in a relatively short interval, various surveys addressed to the elderly population have revealed multiple negative and aggravating effects on their emotional or mental balance, wellbeing, and quality of life. The results of these surveys can constitute part of a new framework able to support more efficiently elderly patient-centered management, adapted to the dysfunctions and challenges brought by this new pandemic in a society that was already in an accelerated aging.

The proposed procedure for the management of the risk of a mental and emotional long-term decline of the elderly following SARS-CoV-2 infection is based on an integrated and complex system of information, recent research results, and knowledge regarding the mental and emotional health of elderly patients. The proposed procedure is scalable and perfectible through the periodic analysis of the results of the management of mental and emotional health, through sequential reiteration in the case of identifying inappropriate results, and through longitudinal studies for the assessment of the efficiency and impact of the management. The integration of RMDSs in multiple phases of the procedure gives it new capabilities that support the approach to mental and emotional health through personalized, preventive, and proactive healthcare services. An RMDS such as the RO-SmartAgeing system is a concrete example of the benefits that non-intrusive monitoring of the health status, lifestyle, and daily activities of an elderly patient, combined with support services for sustaining some social relationships and an active and independent life, with a real-time dynamic link to the medical staff, can bring to the efficiency of the management of the elderly’s mental and emotional health. The RO-SmartAgeing system is to be continuously developed and completed with new functionalities based on new information and requirements resulting from new research, surveys, and medical specifications aimed at remote monitoring of elderly patients.

## Figures and Tables

**Table 1:** Short synthesis of some projects that reflect the state-of-the-art in Romania in the field of research aimed at the remote health monitoring of the elderly.

“Smart assistant to prevent and detect cognitive decline, promote cognitive function and social inclusion among older adults (ReMember-Me)” Project [51]	
Brief presentation	The solution developed in this project aims to monitor, detect, and prevent the cognitive decline of the elderly. It comprises a robot, computer games, and sensors for gathering data on the seniors’ status. A monitoring platform is also provided for connecting the elderly with their caretakers and other seniors.
Bottlenecks	It focuses mainly on cognitive issues, without correlating them with other current or potential co-morbidities of the elderly.
Contribution of RO-SmartAgeing system to the progress beyond the state-of-the-art in Romania	The RO-SmartAgeing system provides a framework and facilities for a broad modern approach to the health management of the elderly. The medical history is corroborated and continuously updated with information and data gathered through the RO-SmartAgeing smart environment in real time. The empowerment and engagement of seniors in the management of their health status are strongly taken into consideration and implemented in various functionalities.
**“Red-Button—Integrated services of socio-medical care at home monitored through the telecare system” Project [52]**	
Brief presentation	The project aimed to develop an innovative system for the teleassistance of single elderly persons in Romania. The main device used in this system is a smart bracelet with an SOS button able to trigger an alert signal to the Emergency Medical Dispatcher. This one receives access to the medical history of the senior patient and triggers a personalized emergency protocol, as well as informs the family.
Bottlenecks	The system is still under-development, and it does not provide enlarged functionalities for evaluating the health status, such as continuous health monitoring.
Contribution of RO-SmartAgeing system to the progress beyond the state-of-the-art in Romania	The solution proposed by the RO-SmartAgeing system provides comprehensive capabilities for personalized health monitoring of the elderly, and it facilitates access to supporting information for an independent, active, and healthy life. It also provides functionalities for the assessment of the current health status of the elderly, as their mental and emotional ones.
**“Inclusive online platform for senior adults (iCan)” Project [53]**	
Brief presentation	The solution developed in this project aims to support seniors in their daily life, increasing their motivation to use digital solutions to connect with their families. Some “smart” games, home delivery assistance, and ordering taxis for elderly users are also available.
Bottlenecks	The focus of this solution is to help and entertain the elderly; health monitoring is a secondary target.
Contribution of RO-SmartAgeing system to the progress beyond the state-of-the-art in Romania	The medical component of the RO-SmartAgeing system is its core part, and it aims to support the elderly, their caretakers, and health professionals to non-intrusively monitor the seniors’ health status and daily activities in an age-friendly environment and to provide functionalities able to offer a broad range of information regarding the evolution of their health. At the same time, the information provided by the support services component assists its users in gaining knowledge about successful and safe aging.
**“Clinically-validated INtegrated Support for Assistive Care and Lifestyle Improvement: the Human Link (vINCI)” Project [54]**	
Brief presentation	The solution provided by the vINCI project is based on some technologies developed by the project partners: a smartwatch, smart shoes, and indoor tracking algorithms. It aims to assist the caretakers and the elderly (as out-patients) with smart care.
Bottlenecks	The main scope of remote health monitoring is gathering data for assessing several factors that influence seniors’ quality of life. The number of monitoring technology is quite small.
Contribution of RO-SmartAgeing system to the progress beyond the state-of-the-art in Romania	The RO-SmartAgeing system comprises more IoT-based devices, thus allowing a larger range of health parameters to be monitored and used for more comprehensive medical assistance support.
**“Smart Big Data Platform to Offer Evidence-based Personalised Support for Healthy and Independent Living at Home (SMART BEAR)” [55]**	
Brief presentation	The solution provided by this project aims to optimize the management of the elderly’s diseases and associated risks. It assesses the quality of life of the elderly and their independence level. It comprises sensors, assistive medical, and mobile devices to gather health parameters able to support independent and healthy living.
Bottlenecks	The provided solution is not personalized for a specific user. The targeted elderly category is restricted to persons aged 67–80 and with a medical history containing at least two health conditions (from a pre-defined five).
Contribution of RO-SmartAgeing system to the progress beyond the state-of-the-art in Romania	While the SMART BEAR project has been developed by 27 European partners, the RO-SmartAgeing system has been developed by a single team from Romania; therefore, its aims were not so broad. Even so, its comprehensive functionalities cover most of the above-mentioned ones. Moreover, the RO-SmartAgeing system provides in one of its components informative support for the elderly, their caretakers, and any person interested in a healthy, independent, and active life and aging. The RO-SmartAgeing smart environment can be personalized according to the elderly’s specificities and health status evolution.
**“Ella4Life, your virtual personal assistant for home and on the road” [56]**	
Brief presentation	The solution developed in this project is based on a mobile solution (that supports the elderly to have an active and healthy life while connecting them with their caretaker and health specialists), an avatar (that assists through speech the elderly in performing daily activities), and specially developed sensor technology (that allows remote health monitoring).
Bottlenecks	Only several chronic diseases are addressed. No alarm triggers are provided. The targeted elderly are supposed to be in quite a good state of mental health.
Contribution of RO-SmartAgeing system to the progress beyond the state-of-the-art in Romania	Even if the RO-SmartAgeing system is not provided with speaking capabilities, and it is intended to be used only indoors, it provides functionalities able to support both the management of primary care and of several age-related diseases, including the mental and emotional health of the elderly. Furthermore, it can be personalized according to the specific needs of the senior, and alarms are triggered in case of an emergency or abnormal event.

**Table 2:** Abbreviations used in this paper.

Abbreviation	Meaning
ADL	Activities of Daily Living
BPM	Blood pressure monitor
ECG	Electrocardiogram
INS	National Institute of Statistics
IRES	Romanian Institute for Evaluation and Strategy
MACE	Mini-Addenbrooke’s Cognitive Examination
MCI	Mild cognitive impairments
MMSE	Mini-Mental State Examination
MoCA	Montreal Cognitive Assessment
RMDS	Remote Monitoring Digital Solutions
RO-SmartAgeing	Non-invasive Monitoring System and Health Assessment of the Elderly in a Smart Environment
WHO	World Health Organization
BPM Core	Withings Blood Pressure Monitor

**Table 3:** A sample of questions used in Romanian surveys.

Name of the Survey	Date	Questions Associated with COVID-19
IRES SURVEY “A month of loneliness” [59]	April 2020	(1) How did you get through the first month of the pandemic? (2) Have you felt any of the following states or feelings: fear of infection or death, fear of a future food crisis
IRES SURVEY “Romanians after 2 years of COVID-19” [60]	27 September–12 October 2021	(1) How worried are Romanians about the current COVID-19 pandemic? (2) When do Romanians think the current COVID-19 pandemic will end? (3) How likely is a new pandemic to occur in the near future? (4) Are Romanians more worried about the pandemic or a war in the region? (5) What changes did the pandemic bring to the lives of Romanians? (6) What have Romanians learned due to the pandemic? (7) What limitations have Romanians experienced due to the pandemic? (8) What would Romanians do when all the restrictions in Romania are lifted?
KANTAR ROMANIA, at the request of the Never Alone—Friends of the Elderly Association [61]	15–18 February 2022	(1) How was the state of physical and mental health affected? (2) Have you felt any of the following states or feelings: fear of loneliness, fear of death? (3) Where did they spend their time during the pandemic?

**Table 4:** Degree of concern of the 65+ population relative to the COVID-19 pandemic.

		Age	Gender		Education			Residence	
		65+	M	F	Low	Average	High	Urban	Rural
Thinking about the Covid pandemic, in which of the following situations do you find yourself?	Very + Quite worried	48%	34%	51%	47%	40%	39%	41%	44%
	Neither worried nor unconcerned	1%	1%	0%	1%	1%	0%	1%	1%
	Not at all worried + Rather worried	50%	64%	48%	51%	58%	60%	57%	54%
	I do not know/I do not answer	2%	1%	1%	1%	2%	0%	1%	2%

## Data Availability

Not applicable.
